# Virtual Reality in Medical Students’ Education: Scoping Review

**DOI:** 10.2196/34860

**Published:** 2022-02-02

**Authors:** Haowen Jiang, Sunitha Vimalesvaran, Jeremy King Wang, Kee Boon Lim, Sreenivasulu Reddy Mogali, Lorainne Tudor Car

**Affiliations:** 1 Lee Kong Chian School of Medicine, Nanyang Technological University Singapore Singapore Singapore; 2 School of Biological Sciences, Nanyang Technological University Singapore Singapore Singapore

**Keywords:** virtual reality, medical education, medical students, virtual worlds, digital health education

## Abstract

**Background:**

Virtual reality (VR) produces a virtual manifestation of the real world and has been shown to be useful as a digital education modality. As VR encompasses different modalities, tools, and applications, there is a need to explore how VR has been used in medical education.

**Objective:**

The objective of this scoping review is to map existing research on the use of VR in undergraduate medical education and to identify areas of future research.

**Methods:**

We performed a search of 4 bibliographic databases in December 2020. Data were extracted using a standardized data extraction form. The study was conducted according to the Joanna Briggs Institute methodology for scoping reviews and reported in line with the PRISMA-ScR (Preferred Reporting Items for Systematic Reviews and Meta-Analyses extension for Scoping Reviews) guidelines.

**Results:**

Of the 114 included studies, 69 (60.5%) reported the use of commercially available surgical VR simulators. Other VR modalities included 3D models (15/114, 13.2%) and virtual worlds (20/114, 17.5%), which were mainly used for anatomy education. Most of the VR modalities included were semi-immersive (68/114, 59.6%) and were of high interactivity (79/114, 69.3%). There is limited evidence on the use of more novel VR modalities, such as mobile VR and virtual dissection tables (8/114, 7%), as well as the use of VR for nonsurgical and nonpsychomotor skills training (20/114, 17.5%) or in a group setting (16/114, 14%). Only 2.6% (3/114) of the studies reported the use of conceptual frameworks or theories in the design of VR.

**Conclusions:**

Despite the extensive research available on VR in medical education, there continue to be important gaps in the evidence. Future studies should explore the use of VR for the development of nonpsychomotor skills and in areas other than surgery and anatomy.

**International Registered Report Identifier (IRRID):**

RR2-10.1136/bmjopen-2020-046986

## Introduction

### Background

Traditionally, medical education comprises both theoretical learning in classrooms and clinical training in hospitals where students are able to gain clinical experience [1]. This is mainly done by means of face-to-face teaching. However, there has been a recent shift to the greater adoption of technology in medical education. This has been accelerated by the COVID-19 pandemic. After it was learned that transmission of COVID-19 is decreased by social distancing, educators were forced to rethink how best to teach students while decreasing face-to-face teaching [2]. To solve this problem, digital education has been proposed as a possible solution to improve medical education. Digital education (also known as electronic education or e-learning) is defined as the act of teaching and learning by means of digital technologies [3]. It is a broad term that encompasses a large number of different modalities, from a simple e-book to complex modalities such as virtual reality (VR), mobile learning, virtual patients (VPs), serious gaming and gamification, and digital skills trainers [4]. Although there is a wide range of digital education tools available, in this scoping review we will be focusing on investigating a single modality—VR.

VR is defined as an educational tool that uses computer technology to create a 3D image or environment that one can interact with in a seemingly real or physical way [5]. VR is a broad concept that has many different tools and applications. VR simulators can be classified into surgical VR simulators, 3D anatomical models, virtual dissection tables, virtual worlds or environments, and mobile VR. Surgical VR simulators consist of an interface connected to mechanical devices or haptic units and can be displayed on any screen but most commonly using a desktop [6]. Surgical VR simulators are most effective at developing users’ technical psychomotor skills, such as for endoscopic surgery, because they can be used repeatedly and require very little time to set up [7]. 3D anatomical models allow users to explore 3D models by manipulating and rotating the model [8]. They are most commonly developed from 2D radiological images using different types of software tools [8]. Virtual dissection tables often overlap with 3D anatomical structures but are distinct in that they allow manipulation to *cut* digital models to reveal cross-sectional images; examples include the Anatomage Table [9]. Virtual worlds are 3D virtual environments based on multiplayer web-based gaming, freeing users from the constraints of location and time. Virtual worlds representing a clinical setting have been used to train emergency personnel on the management of situations involving mass casualties or major incidents [10-12]. Avatars representing patients can be generated to provide a more realistic simulation for the user [13]. Mobile VR refers to VR modalities designed for use on a touch screen mobile phone or tablet; examples include the Touch Surgery app [14].

VR can have diverse application in medical education. It has so far been most commonly used for the development of technical competencies, such as surgical skills, or for developing the ability to visualize anatomy in 3D. Examples of its applications include surgical technique training, the development of 3D visualization skills, and training for procedures such as cardiopulmonary resuscitation (CPR) [15-18]. However, VR can also be used to teach *soft skills* such as empathy and communication skills [13,19,20]. This commonly involves the use of avatars in a virtual world mimicking patients that respond in a certain way so that users can communicate with them [19]. Considering the large range of skills that can be taught with VR, coupled with the widespread reach and convenience of digital education, it holds great potential in the future of medical education.

Given the wide array of tools available in the VR toolbox and the diverse areas in which VR can be applied, there is a need to systematically identify the current VR applications used in medical education, as well as to identify any gaps in the current research of VR in medical education as reported in the literature. Although there are reviews aiming to map different applications of VR used in other types of health care education such as nursing and dentistry education, there seem to be none focusing on medical students’ education [21,22]. Existing systematic reviews on VR in medical education mainly focus on assessing the effectiveness of VR within surgical disciplines, more specifically laparoscopic surgery and neurosurgery [23,24]. This scoping review aims to have a much broader focus by mapping out the extent of VR applications, rather than focusing on the effectiveness of VR in a specific subject.

### Objective

The objective of this scoping review is to identify the different VR tools and applications in undergraduate or preregistration medical education as reported in the literature. We also aim to identify any gaps in the existing literature and provide suggestions for future research on the use of VR in medical education.

## Methods

### Overview

The scoping review was conducted in accordance with the Joanna Briggs Institute methodology for scoping reviews [25], which comprises the following six stages: (1) identifying the research question; (2) identifying relevant studies; (3) study selection; (4) charting the data; (5) collating, summarizing, and reporting the results; and (6) stakeholder consultation. The results were reported in line with the PRISMA-ScR (Preferred Reporting Items for Systematic Reviews and Meta-Analyses extension for Scoping Reviews) [26]. The protocol was registered on the Open Science Framework [27].

### Stage 1: Identifying the Research Question

The objective of this scoping review is to outline the different VR modes available and the applications of VR in undergraduate or preregistration medical education. In line with the objectives of this scoping review, we have developed the following research questions:

1. How is VR used in undergraduate or preregistration medical education?
2. What are the main features of the VR applications in undergraduate or preregistration medical education?
3. What VR tools are available for undergraduate or preregistration medical education?
4. To which aspects of undergraduate or preregistration medical education has VR been applied?

### Stage 2: Identifying Relevant Studies

A comprehensive search of the literature was performed using the following electronic databases: MEDLINE (Ovid), Embase (Elsevier), Cochrane Central Register of Controlled Trials (Wiley), and Education Resources Information Centre (Ovid). As a first step, a limited search using keywords was conducted in MEDLINE. The search strategy was piloted to check the appropriateness of the keywords and databases. In all retrieved papers, an analysis of the words contained within the title and abstracts as well as index terms was performed to develop a full search strategy. Thereafter, a second search using all the identified keywords and index terms was performed across all databases in December 2020. Finally, the third step included screening of the reference lists of all studies selected for this scoping review to look for additional sources. The complete search strategies for all databases can be found in Multimedia Appendix 1. The initial MEDLINE search strategy was developed with the help of a medical librarian experienced in the field. The search period ranged from 2010 to the present. We chose to start from 2010 because most literature pertaining to VR for education was published in recent years, as shown by our previous work in this area [28]. The capabilities of digital technology and VR have also changed substantially over time. We searched for literature in the English language only. All references identified were imported into the reference manager software, EndNote X9 (Clarivate). The references from different electronic databases were combined and any duplicate records removed.

### Stage 3: Study Selection

The study selection followed a two-step screening process, which consisted of a title and abstract screening, followed by a full-text review. In both steps, 2 independent reviewers (JHW and SV) screened the articles against the eligibility criteria. Any disagreements were discussed, and if no consensus could be reached, a third reviewer (BMK) was consulted. We considered eligible studies based on the criteria presented in Textbox 1.

The first step involved the screening of the title and abstract of the studies using EndNote X9. To qualify for the full-text scan, the title and abstract had to (1) focus on the use of VR for educational use only and (2) have medical students as the target population. VPs, that is, computer-generated programs that simulate real-life clinical scenarios, can also be delivered in a VR format. In this scoping review, we included VR-based VPs. We also included studies on VR-based serious gaming education. Augmented reality (VR superimposed onto the real-world environment) [22] and mixed reality (mixing of both virtual and digital elements, allowing one to interact with both simultaneously) [29] are distinct entities that make use of VR and are not classified as VR. Studies focusing solely on mixed reality or augmented reality were excluded from this review.

**Figure 1:**
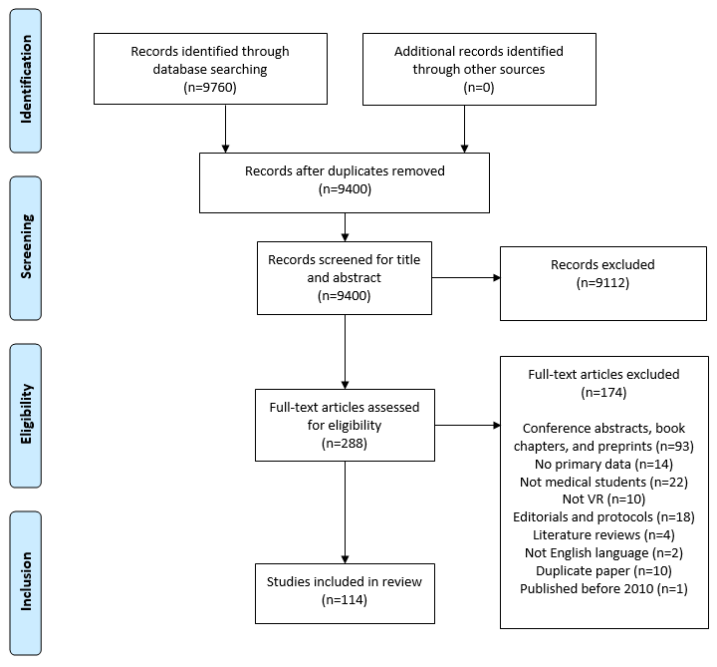
Flow diagram of the literature search and study selection process. VR: virtual reality.

We considered all primary studies, including experimental, observational, and qualitative study designs. Systematic reviews and meta-analyses were also considered. The full texts of the included studies were retrieved and their citation details imported. Studies excluded at this stage are described in Figure 1. This process followed the PRISMA (Preferred Reporting Items for Systematic Reviews and Meta-Analyses) guidelines [30], and 2 review authors (JHW and SV) verified the final list of included studies.

**Full inclusion and exclusion criteria.**

**Inclusion criteria**

- Studies on undergraduate or preregistration medical students in any geographical setting
- Studies involving the use of virtual reality together with another modality such as immersive virtual reality, virtual reality–based serious gaming, and virtual reality–based virtual patients
- All primary studies, regardless of study design, and relevant systematic reviews

**Exclusion criteria**

- Studies focusing only on virtual patient simulation, augmented reality, mixed reality, or serious gaming, without any involvement of virtual reality
- Studies published before 2010
- Studies in languages other than English
- Opinion pieces, viewpoints and conceptual frameworks, and conference abstracts

### Stage 4: Charting the Data

Relevant data were extracted from all included studies by 2 independent reviewers (JHW and SV). A structured data recording form developed by the reviewers was used and the information recorded using Microsoft Excel 2013. The full data extraction form can be found in Multimedia Appendix 2. The data extraction tool was piloted and revised as necessary during the process of extracting data from each study. Any disagreements that arose between the reviewers were resolved through discussion, and a third review author (BMK) acted as an arbiter when disagreements could not be resolved. We contacted the study authors for any missing or incomplete data.

### Stage 5: Collating, Summarizing, and Reporting the Results

To characterize and summarize the results, a map of the data extracted from the included papers was presented in a diagrammatic or tabular form. In alignment with the objectives of this study, we provided an overview of the target participants, content of VR programs, types of studies included, and the context of each included study. The tabulated and charted results were accompanied by a narrative summary, which described how the results met the objectives and aims of this scoping review. We reported the findings in line with the PRISMA-ScR checklist [26]. Using the gap identification process, we detected areas where there was a paucity of data on VR content and its application in undergraduate or preregistration medical education.

We classified VR modalities based on the extent of immersion or interactivity. Immersion can be defined as the sensation of being disconnected with reality [31] or the amount of *presence* experienced by the user due to the illusion rendered by the VR modality [32]. The level of immersion is largely dependent on the number of senses the user uses to interact with the VR environment: the more the senses used, the more immersive the VR environment is said to be. This reflects the system’s technical capabilities: the greater the number of sensorimotor contingencies the system has, the more immersive it will seem [33]. VR has generally been classified into two levels of immersion: immersive VR and nonimmersive VR. Fully immersive VR is defined as VR combined with devices that allow the user to visualize the recorded image in 3D in their entire field of vision and detect eye motions and leap motions of the hands. Nonimmersive VR involves computer-generated experiences on a desktop with which the user interacts by using a mouse [34]. For this study, we will define a third entity, *semi-immersive VR*, which does not fall into either of the 2 categories (eg, head-mounted devices that capture eye motions but do not capture hand motions and desktop-based VR, which provides tactile feedback).

Interactivity in VR refers to the extent to which the user can influence the content or form of the VR environment [32]. This can be classified into low, moderate, or high levels of interactivity. A low level of interactivity simply allows the user to choose information, such as using a mouse to select options that display different anatomical models. A moderate level of interactivity allows the user to add or delete objects in the VR environment, such as a virtual dissection tool that allows users to add or delete various anatomical structures individually. A high level of interactivity refers to when the VR environment responds appropriately to the user’s input, such as using a joystick to manipulate the VR environment in a surgical simulator.

### Step 6: Stakeholder Consultation

A stakeholder consultation was undertaken on August 12, 2021, with the aim of discussing and improving the presentation of our findings. No ethics approval was required as per Nanyang Technological University ethics board guidance. The stakeholder consultation consisted of a 1-hour-long web-based seminar. The audience comprised 18 researchers in the fields of medical education, digital health professions education, and health service research, as well as educators. The stakeholders were invited to share any comments, questions, or suggestions in relation to our study. In addition, we also specifically asked them to share their views on the most important aspects of our findings for researchers and educators, recommendations for future research, and suggestions on any other research in the field of VR or medical education that we should take note of. We have analyzed and presented our findings in this manuscript in line with the information collated through this stakeholder consultation.

## Results

### Included Studies

Our searches identified a total of 9400 studies after duplicates were removed, of which 288 (3.06%) were selected for full-text review. Of these 288 studies meeting the criteria for full-text review, 174 (60.4%) did not meet the inclusion criteria, resulting in 114 (39.6%) studies being included in this scoping review (Figure 1).

### Study Characteristics

Of the included studies, most studies were either randomized controlled trials (RCTs; 47/114, 41.2%) or other experimental design studies (eg, before-and-after and cross-over studies; 49/114, 42.9%). Of the 114 studies, 14 (12.3%) were cross-sectional studies [35-49], 3 (2.6%) were case series or case studies [42,50,51], and 1 (0.9%) was a meta-analysis that examined the effectiveness of 3D anatomical models in teaching anatomy [52], which found that 3D anatomical models yielded significantly better results for user satisfaction and perceived effectiveness compared with conventional 2D teaching methods. An overview of the study characteristics is provided in Table 1.

Among the 96 RCTs and experimental studies included, 50 (52%) compared VR against a traditional learning method (eg, box trainer and video-based lectures), 27 (28%) evaluated VR modalities by changing another variable (eg, VR vs VR with warm-up and VR with guidance vs no guidance) [9,14,48,49,53-94], 14 (15%) did not have any intervention (eg, before-and-after studies and learning curves) [95-109], and 5 (5%) compared a VR modality against another type of VR modality (eg, LapSim vs ProMIS) [110-113].

Of the 114 studies, 30 (26.3%) were from the United States, 11 (9.6%) each from the United Kingdom and Germany, 9 (7.9%) each from Canada and Denmark, and 13 (11.4%) from Asia. Other countries were uncommon, with notably no studies being published from Africa or any low-income country.

Ethics approval was mentioned in 61.4% (70/114) of the studies, and the source of funding was mentioned in 40.4% (46/114) of the studies. Among the 46 studies that received funding, 19 (41%) received funding from the university, 12 (26%) received charitable funding, 9 (20%) received government-backed funding, and 6 (13%) received private funding.

**Figure 2:**
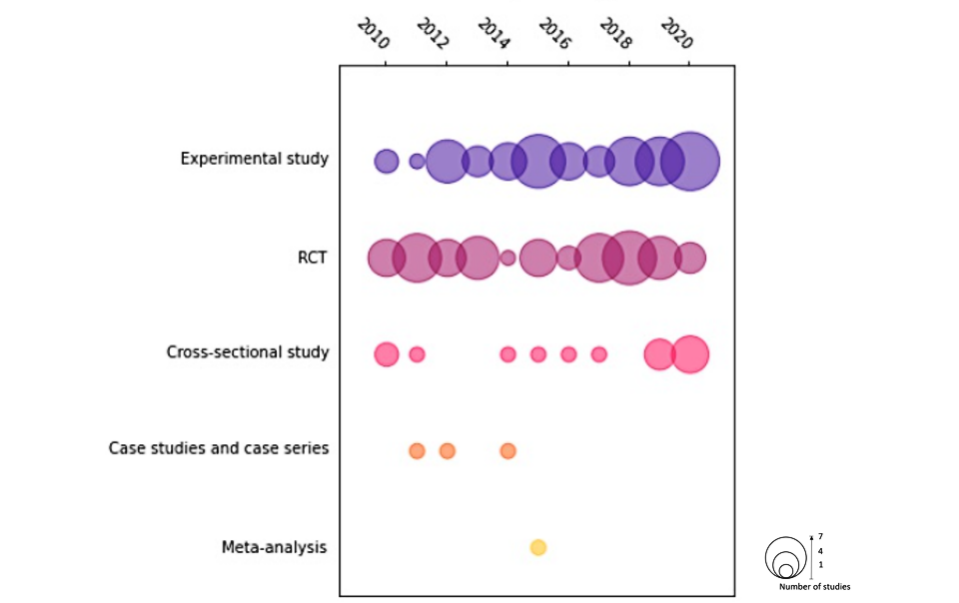
Publication frequency by year of the articles included in this study. RCT: randomized controlled trial.

There was generally an increase in frequency of publication from 2010 to 2020, with 7.9% (9/114) of the studies published in 2010 and 17.5% (20/114) of the studies published in 2020 (Figure 2).

On the basis of our review of the literature on VR in medical students’ education, we categorized the findings from the included studies as follows: (1) students, (2) VR modalities, (3) development, (4) input and output devices, (5) extent of immersion and interactivity, (6) subjects taught, (7) teaching strategies, and (8) assessment methods. These categories will be explored next.

**Table 1:** Characteristics of included studies (N=114).

Domain and feature		Values, n (%)
**Study design**		
	Randomized controlled trial	47 (41.2)
	Experimental (eg, cross-over and before-and-after studies)	49 (42.9)
	Cross-sectional studies	14 (12.3)
	Cases studies and case series	3 (2.6)
	Meta-analysis	1 (1.1)
**Location (by country)**		
	United States	30 (26.3)
	Germany	11 (9.6)
	United Kingdom	11 (9.6)
	Canada	9 (7.9)
	Denmark	9 (7.9)
	Others	44 (38.6)
**Number of students**		
	0-50	76 (66.7)
	51-100	20 (17.5)
	>100	18 (15.8)
**Year of study of students^a^**		
	1	31 (27.2)
	2	29 (25.4)
	3	26 (22.8)
	4	23 (20.2)
	5	19 (16.7)
	6	19 (16.7)
**Study setting**		
	University	108 (94.7)
	Hospital	6 (5.3)
**VR^b^ modalities used**		
	Surgical VR simulator	69 (60.5)
	3D anatomical model	14 (12.2)
	Virtual dissection table	4 (3.5)
	Virtual worlds	21 (18.4)
	Mobile VR	4 (3.5)
	Others	2 (1.8)
**Mode of access**		
	Commercial product	84 (73.6)
	Developed in-house	30 (26.3)
	Both commercial and in-house elements	5 (4.4)
**Input devices**		
	Haptic tools	71 (62.2)
	Mouse	21 (18.4)
	Touch screen	8 (7.4)
	Game controllers	5 (4.4)
	Joysticks	2 (1.8)
	VR gloves	2 (1.8)
	Headset	4 (3.5)
	Stereoscopic glasses	1 (0.9)
**Delivery devices**		
	Screen	100 (87.7)
	Headset	13 (11.4)
	3D projector with stereoscopic glasses	1 (0.9)
**Extent of immersion**		
	Fully immersive	20 (17.5)
	Semi-immersive	68 (59.6)
	Nonimmersive	26 (22.8)
**Extent of interactivity**		
	High	79 (69.3)
	Moderate	19 (16.7)
	Low	16 (14)
**Subject taught^a^**		
	Surgical psychomotor skills	71 (61.4)
	Anatomy	21 (18.4)
	Clinical management^c^	16 (14)
	Radiology	4 (3.5)
	Nonsurgical psychomotor skills	3 (2.6)
	Communication	3 (2.6)
**Mode of teaching**		
	Self-directed	71 (62.3)
	Guided	42 (36.8)
	Not available^d^	1 (0.9)
**Duration of teaching**		
	<1 day	35 (30.7)
	1 day to 1 month	28 (24.6)
	1-6 months	16 (14)
	6-12 months	8 (7)
	>1 year	4 (3.5)
	Not specified	23 (20.1)
**Timing of assessment**		
	Immediate	96 (84.2)
	Delayed	17 (14.9)
	Not available^d^	1 (0.9)
**Individual or group delivery^a^**		
	Individual	97 (85.1)
	Individual and group	7 (6.1)
	Group	9 (7.9)
	Not available^d^	1 (0.9)

^a^Percentages do not add up to 100% because of overlap among the included studies.

^b^VR: virtual reality.

^c^Examples include cardiopulmonary resuscitation, pediatric respiratory management, clinical presentation, and trauma management.

^d^The systematic review did not investigate any mode of teaching.

### Students

Of the 114 studies, 76 (66.7%) involved ≤50 students, 20 (17.5%) involved 51-100 students, and 18 (15.8%) involved >100 students. All years of study of medical students were generally well represented, with a slight tendency to include lower-year medical students.

Most of the studies (108/114, 95.7%) took place in a university setting, with the remainder (6/114, 5.3%) taking place in a hospital setting [111,114-118].

### VR Modalities

**Figure 3:**
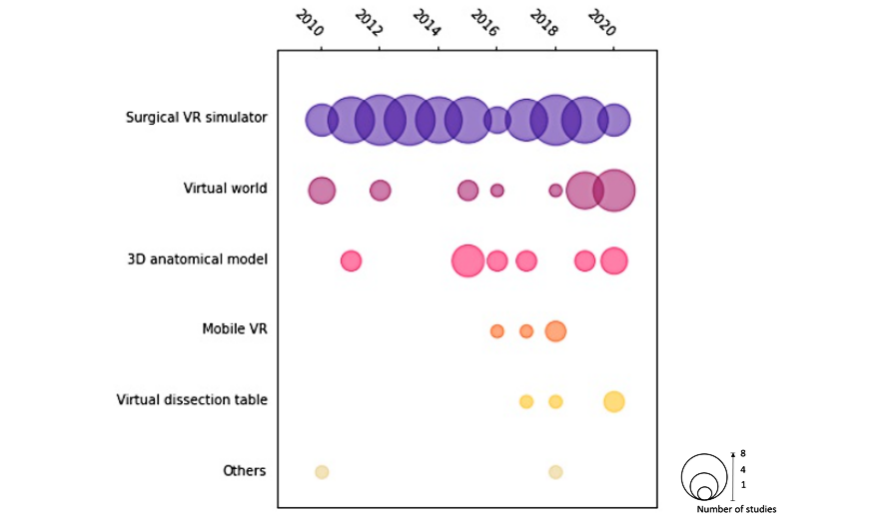
Number of papers of each VR modality published by year. VR: virtual reality.

Of the 114 papers, 69 (60.5%) concerned surgical VR simulators [36,37,42,46,47,51,53,55-57,59,66,67,73-75,79,81-84,86,88,90-94,96, 98-100,103,105,107-138], 20 (17.5%) used virtual worlds or virtual environments [39-41,43, 44,48,50,58,69,70,76-78,101, 139-145], 15 (13.2%) used 3D anatomical models, 4 (3.5%) used virtual dissection tables [9,62,97,127,146], 4 (3.5%) used mobile VR [14,63,65,147], 1 (0.9%) examined the use of a virtual palpation simulator, and 1 (0.9%) used a virtual ultrasound simulator (Figure 3).

**Figure 4:**
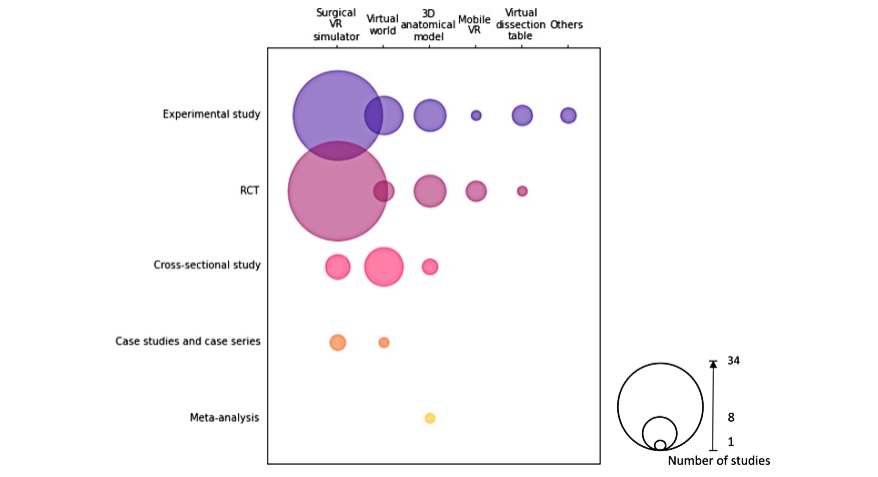
VR modality described against study design. RCT: randomized controlled trial; VR: virtual reality.

Most surgical VR simulators were evaluated using either RCTs (34/69, 49%) or experimental studies (29/69, 42%). Similarly, most 3D anatomical models were also evaluated by either RCTs (6/15, 40%) or experimental studies (6/15, 40%). Virtual worlds were mainly evaluated using experimental studies (8/20, 40%) or cross-sectional studies (8/20, 40%). Mobile VR was mainly evaluated through RCTs (3/4, 75%), whereas virtual dissection tables were mainly evaluated through experimental studies (3/4, 75%; Figure 4).

Among the studies using surgical simulators, approximately one-third (22/69, 32%) [42,53,59,74,82,86,89,91, 94,105,110,111,113, 119,122,123,128,130, 132,136,148] used some version of LAP Mentor [149]. There were also a notable number of studies using ARTHRO Mentor [150] (7/69, 10%) [36,56,66,110,112,120,121], Eyesi Virtual Simulator (3/69, 4%) [37, 51,133], da Vinci Surgical Simulator (4/69, 6%) [90,96,117,118], dV-Trainer (4/69, 6%) [82,88,103,126], VBLaST suturing simulator (3/69, 4%) [49,84,99], and SimSurgery (3/69, 4%) [106,125,131]. Other surgical VR simulators were uncommon.

Among the studies using 3D anatomical models, most (11/15, 73%) were developed in-house by the authors themselves, with the exception of some studies in which commercial products were used. They include Surgical Theater’s Precision VR visualization platform, which is a commercial product used to visualize cerebrovascular anatomy using a controller [35], and DIVA, which is a 3D VR platform used for craniofacial trauma education [151].

Among the 20 studies involving virtual worlds, 15 (75%) were developed in-house, whereas the remaining 5 (25%) used virtual worlds that are commercial products, including products such as MicroSim [58], Body Interact [141], Otago virtual hospital [50], a beta version of CPR VR learning software [70], and Medical Realities VR [87].

Among the 4 studies involving the use of virtual dissection tables, 2 (50%) used the Anatomage Table [9,146], 1 (25%) used the Sectra Virtual Dissection Table [97], and 1 (25%) used the VH Dissector Pro [62].

Among the 4 studies involving the use of mobile VR, 3 (75%) used the Touch Surgery app, a mobile surgical training platform [14,65,152], and 1 (25%) used the aVOR app, a teaching, training, and testing tool for the vestibulo-ocular reflex system and its disorders [63].

The most common commercial products described in the literature are summarized in Textbox 2.

**Common commercial virtual reality (VR) products used in the included studies.**

**VR modalities and types of tools used**

- Surgical VR simulators
  - LAP Mentor
  - ARTHRO Mentor
  - Eyesi Virtual Simulator
  - da Vinci Surgical Simulator
  - dV-Trainer
  - VBLaST suturing simulator
- Virtual worlds
  - MicroSim
  - Body Interact
  - Otago virtual hospital
- Virtual dissection tables
  - Anatomage Table
  - Sectra Virtual Dissection Table
  - VH Dissector Pro
- Mobile VR
  - Touch Surgery app
  - aVOR app

### Development

Of the 114 studies, 35 (30.7%) used a VR modality that was developed in-house. The information used in development can be broadly classified into four different categories: development of 3D anatomical models, virtual worlds, VPs (clinical scenarios), and probes and haptic devices.

Of the VR modalities developed in-house, 37% (13/35) were 3D anatomical models. Of these 13 studies, 12 (92%) developed 3D anatomical models that used some form of transverse 2D images in their development, either through magnetic resonance imaging or computed tomography images or transverse cross-sectional images of human cadavers. The information was imported into a software program that could convert the 2D images into 3D models (eg, Mimics, Macromedia Flash, and After Effects) [153]. Any defects or irregularities would then be smoothened out manually by means of the software. The model would then be imported into VR platforms (eg, Unreal Engine VR platform and HTC Vive software development kit) where it could be displayed on various VR modalities. The remaining study used 2D diagrams and anatomical descriptions from textbooks and journals [85].

Of the VR modalities developed in-house, 43% (15/35) were virtual worlds. Virtual worlds followed a somewhat similar development pathway but differed in terms of the software and information used and the outcomes of development. Whereas 3D anatomical models aim to produce a model that can be manipulated by the user on a screen, the structures in 3D worlds do not require as great a degree of manipulation; they mainly involve the users exploring the models and interacting with other users through an avatar, and this influences the software used in development. Structures in virtual worlds were mainly built from standard building shapes such as blocks, spheres, and tubes and are called *primitives* or *prisms* [40]. Of the 15 studies focusing on virtual worlds, 5 (33%) used the Second Life platform to develop the virtual world structure, whereas 3 (20%) used Amira. Once the virtual world was completed, users would download the program on a desktop and have to learn the interface before accessing the resources in the world.

Of the 15 studies that examined virtual worlds, 4 (27%) used VPs [41,43,50,101]. The VPs used in the simulations were designed with a predefined set of responses to questions asked by the user. These responses are usually written onto a script and programmed into the VP. In addition, the modality in the study by Guetterman et al [101] used intelligent VPs that can detect body motion as well as facial expression and speech and then modify their responses appropriately and thus can also train the user in nonverbal behaviors. Another modality incorporated a dynamic analysis process where the program was able to compare the user’s performance with that of peers and expert choices and provide feedback in real time [41]. The study by Kleinert et al [43] also noted the importance of incorporating established game design elements to promote long-time motivation, such as a reward system.

Of the 35 studies that used a VR modality that was developed in-house, 7 (20%) examined the development of probes and haptic devices. Of these 7 studies, 5 (71%) [48,57,112,145,154]. used a surgical VR simulator that was developed in-house and described the processes involved in fine-tuning the VR simulator for students’ use. The probes and haptic devices used in these studies were mainly commercially acquired, but the fine-tuning of these devices was performed in-house. This was mainly done by examining the learning curves of the VR simulators and determining the accuracy and reproducibility of the probes and haptic devices involved. This allowed researchers to determine the optimal sensitivity of the probes and the optimal duration of training. Of the 7 studies, the remaining 2 (29%) described the development of novel VR simulators with unique haptic devices. The study by Karadogan et al [104], which described the use of a virtual palpation simulator, was mainly focused on designing a haptic device needed to quantify the amount of force needed to be applied to the haptic device to instill a change in the VR environment. This was measured using the Weber fraction, which is defined as the ratio of the minimum difference that a person can distinguish to the standard intensity of the stimulus in a sensory modality. The second study involved designing a virtual ophthalmoscope that used a cylindrical plastic canister to view photos of the fundus using the ophthalmoscope [46]. The study also focused on adopting *gamification* to improve students’ use of the simulator.

In all the studies with VR modalities developed in-house, the main persons in charge of development of the VR modality were the authors themselves. In addition, 60% (21/35) of the studies mentioned the inclusion of additional experts such as ear, nose, and throat (ENT) surgeons; radiologists; or other specialists in the area of interest to help with validation of the study [37,43,44,46,50,58,63-66,71,72,77,78,85,88,120,121,133, 147,155].

There were generally very few frameworks or theories applied in the development of VR simulators in medical education. Among the 35 studies that used a VR modality developed in-house, only 3 (9%) involved any frameworks or theories when developing the VR modality. The study by Lorenzo-Alvarez et al [78], which investigated the design of game-based learning in virtual worlds, used theories on human learning, especially behaviorism, cognitivism, and constructivism. The study by Makransky et al [44], which investigated the feasibility of developing a desktop VR laboratory simulation on the topic of genetics, used feedback based on the cognitive theory of multimedia learning. The study by Hayward et al [41], which designed a novel tool for teaching diagnostic reasoning, used script theory, which states that the clinician draws upon prestored reasoning pathways in the form of illness scripts or *profiles* when navigating new patient encounters.

### Input and Output Devices

Of the 114 studies, 71 (62.2%) used a haptic surgical tool as their input interface [36,37,42,47,49,51,55,56,59,66,67,73-75,79, 81-84,88-91,94,96,99,100,102-113,115-126,128,129,131-138,148,156] (Figure 4). The next most common input device was a mouse (21/114, 18.4%) [8,38-41,43-45,50,54,58,61,62,64,68,71,76-78, 80,92,101,139-142,144,151,157-164], followed by touch screen (8/114, 7%) [9,14,63,65,97,127,146,147,165,166] and handheld game controllers (5/114, 4.4%) [35,57,87,153,155]. Of the 114 studies, 4 (3.5%) [46,143,144,167] used headset devices such as Oculus Rift VR System, Google Cardboard version 1, and RITECH II, whereas 2 (1.8%) used joysticks [69,168] and 3 (2.6%) [70,95,169] used VR gloves. The study by Kockro et al [72] used stereoscopic glasses as the input device (Figure S1 in Multimedia Appendix 3).

The delivery devices used include the use of screens in most of the studies (100/114, 87.7%) [8,9,14,35-47,49-51,53-56, 58,59,61-69,71,73-79,81,83-86,88-91,93-95,97-104, 106-117,119-129,131-138,140-144,146,147,151,156-159, 161-166,168,170,171], headset (13/114, 11.4%) [48,57,70,80, 82,87,92,95,96,105,118,145,153,155,167,172], and 3D projector with stereoscopic glasses (1/114, 0.9%) [72] (Figure S2 in Multimedia Appendix 3).

### Extent of Immersion and Interactivity

Most of the studies included modalities that were of high interactivity (79/114, 69.3%) [35-37,42,43,45-51,55-57,59,66, 67,70,73-75,79,81-84,86-91,93,94,96, 98-100,102,103,105-113,115,116,118-129, 131-135,137,143,145-147,156,168,171,173,174], whereas 16.7% (19/114) of the studies [9,38-41,62-64,68, 69,76-78,97,104,140,141,151,153,159,161,162,165,169] included modalities that were of moderate interactivity and 14% (16/114) of the studies included modalities that were of low interactivity [8,14,44,54,58,61,65,71,72,80,85,87,92,101, 142,155,157,158,160,163,164, 167,172,175] (Figure S3 in Multimedia Appendix 3).

In terms of immersion, most of the studies included modalities that were semi-immersive (68/114, 59.6%) [36,37,42-44,47,49,51,53,55,56,59,61,66,67,69,73,75,81,83,84,86, 88,89,91,93,94,96,99-101,103,106,108,109,111-117,120,121,123-129, 131-139,142,146,156,166,168-170,176], followed by nonimmersive (26/114, 22.8%) [8,9,14,38,39,41,45,50,54, 58,62,63,65,68,71,72,76-78,85,97,101,140,141,147,157-159, 161,162,164,165,171] and fully immersive (20/114, 17.5%) [35,46,48,57,70,74,80,82,87,90,92,110,118,119, 145,151,153,155,167,172] (Figure S4 in Multimedia Appendix 3).

Of the 114 studies, 14 (12.3%) were both of high interactivity and fully immersive [35,46,48,57,70,74,82,90,105,110, 118,119,145,154]. Of these 14 studies, 9 (64%) involved the development of surgical psychomotor skills: 6 (67%) for laparoscopy [74,82,105,110,118,119] and 1 (11%) each for neurosurgery [35], orthopedics [57], and robotic surgery [90]. Of the 14 studies, 4 (29%) were relating to clinical management: 3 (75%) for pediatrics [48,145,154] and 1 (25%) for CPR [70]. The study by Wilson et al [46] was relating to ophthalmology anatomy. Keeping to definitions of immersion and interactivity, the VR modalities used in these studies allowed the user to visualize the environment, had motion-tracking capability, and allowed the user to manipulate the VR environment in real time.

### Subjects Taught

From the studies, six broad subjects taught were identified: surgical psychomotor skills, anatomy, clinical management of various conditions, radiology, communication, and nonsurgical psychomotor skills.

The most common subject taught was surgical psychomotor skills, with 62.3% (71/114) of the studies including it as a subject taught [14,35-37,47,49,51,53,55-57,59,65-67,73-75, 79,81-84,86-91,93,94,96,99,100,103,105-126,128-138,147,148]. The second most common subject taught was anatomy, with 18.4% (21/114) of the studies including it as a subject taught [9,35,38,40,46,52,54,61,62,64,68,71,72,80,85,92,97,146,153,155,166], followed by 14% (16/114) of the studies including clinical management of various conditions as one of the subjects taught [39,41,43-45,48,50,58,63,69,70,140,141,145,154,167], 3.5% (4/114) of the studies including radiology as a subject taught [64,76-78], 2.6% (3/114) of the studies including nonsurgical psychomotor skills as a subject taught [42,102,104], and 2.6% (3/114) of the studies including communication as one of the subjects taught [44,50,101] (Figure S5 in Multimedia Appendix 3).

Of the 114 studies, 4 (3.5%) taught a combination of subjects. Of these 4 studies, 2 (50%) combined the teaching of clinical management and communication [44,50], 1 (25%) combined anatomy with radiology [64], and 1 (25%) combined anatomy with the development of surgical psychomotor skills [35].

With a focus on surgical psychomotor skills, most of these VR modalities involved the handling of laparoscopic surgeries (39/71, 55%). Of these 39 studies, 23 (59%) explored basic laparoscopic handling skills [49,59,67,74,79,81,83,84,86,96,99, 105,108,111,113, 117,118,123,128,130, 131,148] and 16 (41%) explored advanced laparoscopic surgery procedures [75,82,87,89,91,94,106,110,119,122,125,129, 132,134,136,137] such as cholecystectomy, appendectomy, salpingectomy, and Nissen fundoplication.

Of the 71 studies with a focus on surgical psychomotor skills, 7 (10%) involved arthroscopic VR modalities. Of these 7 studies, 3 (43%) provided training in basic arthroscopic skills [112,120,121], 2 (29%) pertained to knee arthroscopy [56,107], 1 (14%) pertained to hip arthroscopy [36], and 1 (14%) pertained to shoulder arthroscopy [66].

Among the remaining 35% (25/71) of the studies that examined surgical psychomotor skills, specific procedures were involved, such as ENT [114-116,124,138], endoscopy [73,93,100,135], ophthalmology [37,51,55,133], robotic surgery [90,103,126], neurosurgery [35,47], orthopedics [57,147], vascular surgery [53,109], microsurgery [14], urology [88], and emergency procedures (chest tube placement) [65].

Among the studies that explored anatomy, the most prevalent topic was neuroanatomy (6/21, 29%) [35,54,62,64,72,155], followed by regional anatomy (5/21, 24%) [9,52,92,97,146], ENT (3/21, 14%) [68,85,166], vascular anatomy (2/21, 10%) [38,61], and specific anatomical structures (5/21, 24%) [40,46,71,80,153].

Of the 16 studies that included clinical management, 4 (25%) included CPR as a management procedure [39,58,70,140], 3 (19%) were on pediatric respiratory management [48,145,154], and 2 (13%) were on general clinical presentation management [41,141], whereas the remaining 7 (44%) were on specific clinical and situational management procedures, including neurological management for benign paroxysmal positional vertigo [63], trauma [69], surgical [43], palliative [167], prenatal genetic screening [44], patient interaction [50], and clinical ethics management [45].

Of the 4 radiology studies, 3 (75%) pertained to general radiology [76-78] and 1 (25%) explored neuroanatomy together with neuroradiology [64].

Of the 114 studies, 3 (2.6%) involved VR training for nonsurgical psychomotor skills, including intravenous cannulation [42], ultrasound manipulation [102], and palpation [104]. Finally, of the 114 studies, 3 (2.6%) pertained to communication training, which included empathy [101], professionalism in clinical context [50], and prenatal genetic screening [44].

### Teaching Strategies

Most of the studies (103/114, 90.4%) were conducted outside of the medical students’ curriculum, whereas 9.6% (10/114) of the studies assessed VR modalities that were incorporated into the curriculum. Among these 10 studies, the most common method of incorporating VR modalities into the curriculum was either by incorporating 3D anatomical models or virtual dissection tables into anatomy education (4/10, 40%) [38,72,97,146] or by incorporating virtual-world scenarios into clinical placements (4/10, 40%) [45,46,48,145], such as training students how to react to different situations that may be difficult to replicate in real life. The remaining 20% (2/20) of the studies incorporated the VR modality in the final year of study to better prepare students before they graduate. The study by De Ponti et al [141] prepared students for the clinical management of cardiovascular, cerebrovascular, trauma, pulmonary, infective, gynecological, gastrointestinal, renal, and metabolic endocrinology clinical cases, and the study by Paschold et al [106] prepared students for handling laparoscopic instruments in retraction of tissue and cystic duct and artery clipping.

More than half of the studies involved students engaging in self-directed learning with the VR modalities they were provided (71/114, 62%) [14,36,37,40,42-47,49,51,53,56,57,65-68,71-76, 78-82,84,85,87-89,91-93,99-105,107,109-112,114-116,118-121, 123,125,126,128,129,131,132,146,148,153,155,166,167]. Of the remaining 43 studies, 42 (98%) [9,35,38,39,41,48,50, 54,55,58,59,61-64,69,70,77,83,86,90,94,96, 97,106,108,113,117,122,124,130,133-138,140,141,145, 147,154] described students engaging in guided teaching sessions with VR use, whereas 1 (2%) did not provide clear description of student guidance [52].

Of the 42 studies with guided VR training sessions, 26 (62%) asked external experts to guide the students in the topic explored through VR [35,38,48,58,59,61-63,86,90,94,97,106,108,117, 122,124,130,133-136,141,145,147,154]. With regard to the external experts, their number and specialty varied greatly. Examples of external experts guiding students in various subjects included experienced surgeons’ demonstration and commentary on laparoscopic surgery [59], an anatomy instructor teaching an anatomy lesson [61], and otorhinolaryngology residents teaching clinical management of benign paroxysmal positional vertigo [63].

Between the self-directed and guided VR trainings, most of the studies incorporated an introductory session where time was allocated for students to become familiar with the VR system they were provided. Among the 71 self-directed studies, 58 (82%) used an introductory session [36,37,42-44,46,47,49,51,53,56,57,65,68,73-76, 78,82,84,85,87-89,91-93,99,100,102,103,105,107,109-112, 114-116,118,120,121,123,125,126,128,129,131,132,146, 153,155,166]. This took on many forms, such as watching demonstration videos [111,112,121], printed instructions [75,112], or live demonstration [65,100]. A few of the studies (9/71, 13%) did not introduce self-directed students to the use of the VR modality [45,66,67,71,72,81,101,104,167]. However, most of the VR modalities used in these studies had guides built into the VR programs.

Of the 71 studies with guided teaching, 36 (51%) incorporated an introduction for the VR modality [35,39,41,48,55,58,59,61-64,69,77,83,86,90,94,96,106,108, 113,117,122,124,130,133-138,140,141,145,147,154], whereas 6 (8%) [9,38,50,54,70,97] did not explicitly state that time was set aside for an introduction to the VR modality. Interestingly, of these 6 studies, 5 (83%) were conducted as part of the medical curriculum. Of these 5 studies, 1 (20%) [9] was conducted over a week. Although the authors did not explicitly set aside time for orientation to the VR modality, there may have been more time available in total for students to get familiar with the VR equipment.

### Duration of Teaching

There was a wide variation in VR use periods in the studies. Hence, they were categorized into the following time periods: <1 day, 1 day to 1 month, 1-6 months, 6-12 months, and >1 year. For studies with duration >1 month, the 6-month threshold was chosen to distinguish between an academic semester and an academic year.

The most common lengths of teaching periods were <1 day (35/114, 30.7%) [38,43-45,50,53,54,64,71-73,75-77,79,80,83, 88,89,91,92,106,107,109,116,118,122,129,134,137,147,153, 155,166,167] and 1 day to 1 month (28/114, 24.6%) [9,37,42,48,49,57-59, 69,74,78,84-86,90, 99,100,104,108,110, 123-126,130,131,145,154].

Fewer studies opted for longer teaching periods. Of the 114 studies, 16 (14%) used teaching periods lasting 1-6 months [36,39,56,63,65,66,81,87,97,105,111,135,136,146,148], 8 (7%) used periods lasting 6-12 months [35,40,67,94,114,120,121,141], and 4 (3.5%) were conducted over periods lasting >1 year [41,70,115,140].

Of the 114 studies, 4 (3.5%) investigated attainment of proficiency over time, and thus a predetermined training duration was not applicable [82,113,128,133], whereas 1 (0.9%) was a meta-analysis, and thus training duration was not applicable either [52]. The teaching period was not specified in 15.8% (18/114) of the studies [14,46,47,51,55,61,62,68,93, 96,101-103,112,117, 119,132,138].

### Delivery of VR Modalities to Individuals or Groups

The studies had variations in the number of students who were taught using 1 VR device. Hence, the studies were categorized into those that used VR modalities that facilitated teaching an individual and those that facilitated group teaching (>1 person). Some VR modalities were more flexible: they allowed for teaching either an individual or a group.

Most of the study designs involved individual students taught using VR modalities (97/114, 85.1%) [14,36,37,42-49,51,53-59,63,65-67,69-71, 73-76,78-82,84-94,96,99-126,128-138, 141,145-148,153-155,166,167]. A few studies used VR teaching modalities for both individual and group teaching (7/114, 6.1%) [35,40,41,61,62,64,68], whereas some used it solely for group teaching (9/114, 7.9%) [9,38,39,50,72,77,83,97,140]. VR delivery was not applicable for 0.9% (1/114) of the studies [52].

There were distinct group sizes that were characteristic of the modality of VR used. Some studies used small teaching groups of approximately 2-4 students [140]. These VR modalities used virtual world scenario-based teaching methods and involved working in small teams for learning. Other studies used classroom-size teaching methods with 20-30 students [38,72]. These studies mainly focused on anatomy teaching with the use of stereoscopic 3D projectors. Finally, some studies incorporated VR modalities that allowed for trainings to be conducted to hundreds of students at once [77]. These VR modalities were characteristically virtual world massively multiplayer online games such as Second Life.

## Discussion

### Summary of Findings

In this scoping review, we mapped out the existing VR modalities used in undergraduate medical education, including the characteristics of the VR modalities, target population, tools used in development, educational elements, and the outcomes measured of each VR modality. We found 114 studies that were relevant to our objective, including 47 (41.2%) RCTs, 49 (42.9%) other experimental study designs, 14 (12.3%) cross-over studies, 3 (2.6%) case studies and cases series, and 1 (0.9%) meta-analysis. Most of the papers were published from Europe or the United States. Approximately half of the papers reported the use of surgical VR simulators, with the next most common being 3D anatomical models and virtual worlds. Other VR modalities such as virtual dissection tables and mobile VR were less common. The included studies usually used haptic tools or a mouse as input devices and a screen as a delivery device. Most of the studies were semi-immersive with a high degree of interactivity. The most common subject taught using VR simulators was surgical skills, and the most common mode of training was self-directed. There was a large variation in the duration of teaching. Most studies reported only a single type of outcome measurement, with the most common being skills outcomes. The timing of assessment was most often immediately after the intervention. Most VR modalities were also designed for individual delivery rather than group delivery.

### Comparison With Existing Literature and Future Recommendations

Although surgical VR simulators, 3D anatomical models, and virtual worlds are relatively well represented in the literature, there is limited evidence on the use of virtual dissection tables and mobile VR. Indeed, there are a number of systematic reviews evaluating the use of surgical VR simulators in health professions education at both postgraduate and undergraduate level, most of which favor VR, especially for nonsimulation training [177-179]. The relative lack of studies on virtual dissection tables and mobile VR could be due to the fact that these VR modalities are more novel and have been reported in the literature only from 2015 onward, as revealed by our search strategy. Furthermore, some popular VR anatomy applications are not assessed in the included studies, such as Complete Anatomy (3D4Medical) [150] and Anatomy.tv (Primal Pictures) [180]. It seems that although a wide variety of VR tools were mentioned in the results, there are other VR tools that may be commonly used but not mentioned in the literature. Future studies should examine the effectiveness of the use of novel VR modalities in different settings, for example, remote, home-based learning, such as in the case of mobile VR modalities.

Most of the studies included in our review did not report, or refer to, educational or behavior frameworks or theories used in the development of VR applications. This has also been observed in studies on other digital modalities used in health professions education [3]. However, explicit use of frameworks or theories for the design of complex interventions such as the use of VR in education has an important role for improving the quality, transparency, and reproducibility of research. Future research should aim to incorporate and report on the adoption of such frameworks in the design of VR applications where possible.

We also observed several studies exploring the development of particular 3D anatomical models and virtual worlds that had a considerable overlap in terms of the process of development. There is a need for stronger collaboration and easier sharing among educators and researchers in this novel field. This could be achieved through a common platform or database of VR medical education tools and insights similar to Radiopaedia for radiology and GitHub for software engineering.

There is a clear lack of studies from low- and middle-income countries. Adoption of VR tools shown to be effective in high-income countries might not be possible in other settings because of context-specific limitations such as lack of financial resources, knowledge, or technology [181,182]. Given the potential that VR has in improving medical education, there is a need for development and evaluation of VR tools that would be specific to low- and middle-income countries.

We also observed a distinct lack of studies focusing on the use of VR for developing *soft skills* such as communication skills or empathy. The manner in which health care professionals communicate with patients is argued to be as important as clinical knowledge but often goes underemphasized [50,101]. VPs in particular can be programmed to respond in different manners depending on the response of the user and offer an exciting opportunity to develop students’ communication skills from the comfort of their own homes. There is also scope for more research exploring the use of VR for nonsurgical skills development.

Immersive VR modalities not only offer a realistic experience to the user, but they also have the additional benefit of spatial understanding [155]. The higher the level of immersion, the greater the spatial understanding, which can result in greater effectiveness of scientific visualization. It also helps to reduce the information clutter wrought by the overlapping icons and controls of 2D environments [21]. However, highly immersive systems can be costly and resource intensive [28]. Most of the studies in this review were semi-immersive in nature, possibly to optimize realism while avoiding high costs. Future studies should explore the use of VR modalities with high immersion. Correspondingly, there is scope for more research on VR delivered through headsets and VR using input devices other than haptic surgical tools or a mouse.

Only a few studies reported on the integration of VR training presented in the study into medical school curricula [35,70,141]. Although VR is being increasingly implemented at medical schools worldwide, the literature reporting its implementation and adoption is scarce. This is coupled with a lack of guidance or information on how best to adopt different VR modalities in the curriculum. There is a need for clear guidance and recommendations with the aim of enabling optimal adoption and harnessing of VR within medical curricula.

### Strengths and Limitations

We performed a comprehensive search of 4 major bibliographic databases in this review. We covered the search period starting from 2010 to include all available studies on VR-based training for medical students’ education. Our screening and data extraction were also conducted in parallel and independently to ensure reliability and reduce bias in our findings. The topic that we explored was also novel, particularly in the context of undergraduate medical education.

This scoping review was limited to studies published in English. Because of the large number of studies on VR, we only focused our research on the use of VR in medical students’ education and thus the use of VR in other health care professionals’ education and training was not captured in this review. Diverse terminology was used to describe VR; therefore, we may not have captured some studies because of the unfamiliar terminology used. In the categorization of the diverse terminology used in the studies, details specific to singular studies may have been lost. Although this review is as comprehensive as possible, there may still be smaller but important studies that were published only as abstracts that were left out of this review. In accordance with scoping review methodology, there was no quality assessment of the included articles; thus, the included studies may be biased or incomplete in terms of some of the information reported.

### Conclusions

The use of VR in medical education is a rapidly expanding and exciting field of study. Current research is mostly centered on surgical VR simulators, virtual worlds, and 3D anatomical models by comparing them with traditional modes of learning. Novel VR modalities such as mobile VR and virtual dissection tables, which are potentially more portable and allow for group learning, respectively, are less well represented in the literature. As an increasing number of medical schools turn toward incorporating VR into their curriculum, there is a need to evaluate these novel VR modalities as well as describe the methods used to incorporate VR into the curriculum. The use of VR to develop communication skills or to allow students to work in a team is also lacking. Most of the VR modalities described are only designed for a single user, which is unlike situations arising in a health care team. The use of modalities such as virtual worlds to create scenarios that require teamwork and communication should be more widely explored.

## Appendix

Multimedia Appendix 1 Search strategies.

Multimedia Appendix 2 Data extraction form.

Multimedia Appendix 3 Supplementary figures.

## Abbreviations

CPR: cardiopulmonary resuscitation
ENT: ear, nose, and throat
PRISMA: Preferred Reporting Items for Systematic Reviews and Meta-Analyses
PRISMA-ScR: Preferred Reporting Items for Systematic Reviews and Meta-Analyses extension for Scoping Reviews
RCT: randomized controlled trial
VP: virtual patient
VR: virtual reality
