# Supplementary material for: Virtual Reality in Medical Students’ Education: Scoping Review
Source: JMIR Med Educ. 2022 Feb 2;8(1):e34860. doi: 10.2196/34860 (PMC8851326; doi:10.2196/34860)
Supplement: Multimedia Appendix 1 [file mededu_v8i1e34860_app1.docx]

*Multimedia Appendix 1: Search strategies*

*MEDLINE (Ovid)*

1. exp Virtual Reality/
2. User-computer interface/
3. Computer Simulation/
4. Computer-Assisted Instruction/
5. (simulat* or virtual realit* or virtual reality simulat* or virtual reality environment* or VRE or three-dimension* or 3D or immersive virtual realit* or immersive VR or HMD* or head-mounted display* or virtual world* or avatar* or virtual patient* or VR room*).mp.
6. 1 or 2 or 3 or 4 or 5
7. exp Education, Medical, Undergraduate/
8. exp Teaching/
9. exp Curriculum/
10. exp Education, Distance/
11. exp Schools, Medical/
12. 7 or 8 or 9 or 10 or 11
13. exp Students, Medical/
14. (medical student* or medical undergraduate*).mp.
15. 13 or 14
16. 6 and 12 and 14
17. limit 16 to yr="2010 -Current"
18. limit 17 to english language

*EMBASE (Ovid)*

1. exp Virtual Reality/
2. User-computer interface/
3. Computer Simulation/
4. Computer-Assisted Instruction/
5. (simulat* or virtual realit* or virtual reality simulat* or virtual reality environment* or VRE or three-dimension* or 3D or immersive virtual realit* or immersive VR or HMD* or head-mounted display* or virtual world* or avatar* or virtual patient* or VR room*).mp.
6. 1 or 2 or 3 or 4 or 5
7. exp Education, Medical, Undergraduate/
8. exp Teaching/
9. exp Curriculum/
10. exp Education, Distance/
11. exp Schools, Medical/
12. 7 or 8 or 9 or 10 or 11
13. exp Students, Medical/
14. (medical student* or medical undergraduate*).mp.
15. 13 or 14
16. 6 and 12 and 15
17. limit 16 to yr=”2010-Current”
18. limit 17 to english language

*ERIC*

1. virtual reality
2. user-computer interface
3. computer simulation
4. computer assisted instruction
5. (simulat* or virtual realit* or virtual reality simulat* or virtual reality environment* or VRE or three-dimension* or 3D or immersive virtual realit* or immersive VR or HMD* or head-mounted display* or virtual world* or avatar* or virtual patient* or VR room*)
6. S1 OR S2 OR S3 OR S4 OR S5
7. education, medical
8. teaching
9. curriculum
10. education, distance
11. medical school
12. S7 OR S8 OR S9 OR S10 OR S11
13. medical students
14. (medical student* or medical undergraduate*)
15. S13 or S14
16. S6 AND S12 AND S15
17. S6 AND S12 AND S15 (**Limiters** - Date Published: 20100101-20201231)
18. S6 AND S12 AND S15 (**Narrow by Language:**- english)

*CENTRAL (Wiley)*

1. MeSH descriptor: [Virtual Reality] explode all trees
2. MeSH descriptor: [User-Computer Interface] this term only
3. MeSH descriptor: [Computer Simulation] this term only
4. MeSH descriptor: [Computer-Assisted Instruction] this term only
5. (simulat* or virtual realit* or virtual reality simulat* or virtual reality environment* or VRE or three-dimension* or 3D or immersive virtual realit* or immersive VR or HMD* or head-mounted display* or virtual world* or avatar* or virtual patient* or VR room*)
6. #1 or #2 or #3 or #4 or #5
7. MeSH descriptor: [Education, Medical, Undergraduate] explode all trees
8. MeSH descriptor: [Teaching] explode all trees
9. MeSH descriptor: [Curriculum] explode all trees
10. MeSH descriptor: [Education, Distance] explode all trees
11. MeSH descriptor: [Schools, Medical] explode all trees
12. #7 or #8 or #9 or #10 or #11
13. MeSH descriptor: [Students, Medical] explode all trees
14. (medical student* or medical undergraduate*)
15. #13 or #14
16. #6 and #12 and #14 (with Cochrane Library publication date from Jan 2010 to Dec 2020)
