# Supplementary material for: Virtual Reality in Medical Students’ Education: Scoping Review
Source: JMIR Med Educ. 2022 Feb 2;8(1):e34860. doi: 10.2196/34860 (PMC8851326; doi:10.2196/34860)
Supplement: Multimedia Appendix 2 [file mededu_v8i1e34860_app2.docx]

*Multimedia Appendix 2: Data extraction form*

| *Category* | *Type of Data* |
| --- | --- |
| *1. Bibliographic information* | 1. Author 2. Title 3. Country 4. Region 5. Income level 6. Ethical approval 7. Funding source |
| *2. Information relating to the inclusion criteria* | 1. Year of study 2. Study design 3. Aim of study 4. Number of students 5. Year(s) of students 6. Number of groups (comparison arms) 7. Type of comparison 8. Study setting |
| *3. Information relating to the study* | 1. Information related to VR modality   1. VR modality used 2. Introduction to VR modality 3. Information used in development 4. Frameworks/theories used in development 5. Name of instrument 6. Method of acquiring 7. Availability to public 8. Mode of access 9. Input and output device 10. Configuration 11. Feedback modality 12. Extent of interactivity and immersion 13. Educators involved in design 14. Educators involved in delivery 15. Technical difficulties   2. Information related to teaching methods   1. Subject and specific subject taught 2. Whether training was part of curriculum 3. Additional educational elements 4. Mode of teaching 5. Duration and frequency of teaching 6. Outcomes measured 7. Mode and type of assessment 8. Post-test timing of assessments 9. Individual/group delivery |
