# Supplementary material for: Virtual Reality in Medical Students’ Education: Scoping Review
Source: JMIR Med Educ. 2022 Feb 2;8(1):e34860. doi: 10.2196/34860 (PMC8851326; doi:10.2196/34860)
Supplement: Multimedia Appendix 3 [file mededu_v8i1e34860_app3.docx]

*Multimedia Appendix 3: Additional figures*

*Figure S1.* Types of input devices used

*Figure S2.* Types of delivery devices used

*Figure S3.* Extent of interactivity

*Figure S4.* Extent of immersion

*Figure S5.* Distribution of all subjects taught
